# Society Meetings

**Published:** 1899-07

**Authors:** 


### SOCIETY MEETINGS.

The American Medical Association during its recent meeting at Columbus elected officers for the current association year as follows: President, Dr. W. W. Keen, of Philadelphia; first vice-president, Dr. C. A. Wheaton, of St. Paul; second vice-president, Dr. E. D. Ferguson, of Troy, N. Y.; third vice-president, Dr. G. M. Allen, of Liberty, Mo.; fourth vice-president, Dr. W. E. D. Middleton, of Davenport, Ia.; secretary, Dr. George H. Simmons, of Chicago; assistant secretary, Dr. J. A. Joy, of Atlantic City, N. J.; treasurer, Dr. H. P. Newman, of Chicago. The next meeting will be held at Atlantic City, N. J., beginning the first Tuesday in June, 1900.

---

The American Neurological Association held its twenty-fifth annual meeting at Atlantic City, June 14-16, 1899. Officers for the ensuing year, who also compose the council, were elected as follows: President, Dr. E. D. Fisher, of New York; vice-presidents, Dr. Morton Prince, of Boston; Dr. James Wright Putnam, of Buffalo; secretary and treasurer, Dr. Graeme M. Hammond, of New York; councillors, Dr. J. H. Lloyd, of Philadelphia; Dr. Joseph Collins, of New York.

---

THE Mississippi Valley Medical Association will hold its twenty-fifth annual meeting at Chicago, October 3-6, 1899, under the presidency of Dr. Duncan Eve, of Nashville. Titles of papers should be sent at once to the secretary, Dr. Henry E. Tuley, Louisville, Ky.

---

THE Niagara Falls Academy of Medicine held its regular monthly meeting at the office of Dr. C. G. Leo Wolf, secretary, Monday evening, June 5, 1899. The newly elected president, Dr. W. A. Scott, delivered his annual address and scientific subjects were then discussed.

---

THE Buffalo Academy of Medicine held meetings during the month of June, 1899, as follows:

*Section on Surgery.*—Tuesday evening, June 6th. Program: Cystitis, J. Henry Dowd; discussion by D. W. Harrington, W. H. Heath, John Parmenter and others.

*Annual Meeting.*—Tuesday evening, June 13th. Program: An address on history of "organo-therapy," by the retiring president, Dr. Roswell Park.

*Section on Pathology.*—Tuesday evening, June 20th. Program: Milk sanitation, Herbert M. Hill; discussion opened by Irving M. Snow; exhibition of specimens.

---

THE Medical Society of the County of Chautauqua will hold its annual meeting at Chautauqua, N. Y., Tuesday, July 11, 1899. The following is the program: 11 a. m. business session; 1 p. m. scientific session. President's annual address, Placenta previa, Dr. M. W. Bemus; Puerperal eclampsia, Dr. J. H. Wiggins; Paper, Dr. J. W. Seaver, Yale University; Paper, Dr. Wm. M. Bemus; Paper, Dr. J. W. Morris; Report of cases. Evening session, illustrated talk by Dr. Wm. S. Bainbridge, of New York. The secretary is Dr. C. A. Ellis, Sherman, N. Y.

The Lake Erie Medical Society held its regular meeting at the Central Hotel, Angola, N. Y., June 30, 1899. The following was the program: Exfoliative enteritis, Dr. W. M. Ward, North Collins. Presentation of cases. Election of officers. Subinvolution of the uterus, by Dr. C. C. Frederick, Buffalo; Colles's fracture, Dr. J. G. Thompson, Angola.

